# Evaluation of the NG-Test CARBA 5 Lateral Flow Assay with an IMP-27-Producing Morganella morganii and Other *Morganellaceae*

**DOI:** 10.1128/spectrum.00793-23

**Published:** 2023-05-18

**Authors:** Nicole J. Tarlton, Meghan A. Wallace, Robert F. Potter, Kailun Zhang, Gautam Dantas, Erik R. Dubberke, Carey-Ann D. Burnham, Melanie L. Yarbrough

**Affiliations:** a Department of Pathology and Immunology, Division of Laboratory and Genomic Medicine, Washington University School of Medicine, St. Louis, Missouri, USA; b Edison Family Center for Genome Sciences and Systems Biology, Washington University School of Medicine, St. Louis, Missouri, USA; c Department of Molecular Microbiology, Washington University School of Medicine, St. Louis, Missouri, USA; d Department of Pediatrics, Washington University School of Medicine, St. Louis, Missouri, USA; e Department of Biomedical Engineering, Washington University School of Medicine, St. Louis, Missouri, USA; f Department of Internal Medicine, Division of Infectious Diseases, Washington University School of Medicine, St. Louis, Missouri, USA; University of Maryland School of Medicine

**Keywords:** Enterobacterales, IMP-27, *Morganella morganii*, NG-Test CARBA 5, carbapenemase, lateral flow assay, metallo-β-lactamase

## Abstract

An isolate of Morganella morganii (MMOR1) that tested susceptible to 3^rd^/4^th^-generation cephalosporins and intermediate to meropenem was characterized as positive for NDM and IMP carbapenemases by NG-Test CARBA 5. Our objective was to further investigate this result, given the inconsistent susceptibility profile and unusual epidemiological profile for our region. The MMOR1 isolate was retested for antimicrobial susceptibilities and characterized for carbapenemase production. MMOR1 tested susceptible to ceftazidime, ceftriaxone, cefepime, aztreonam, and ertapenem, and intermediate to meropenem and imipenem. The isolate tested positive by carbapenem inactivation method (CIM) and CIM+EDTA (eCIM) testing, indicating metallo-β-lactamase production. The isolate tested negative for all carbapenemase genes on Xpert Carba-R, but positive for IMP on repeat testing of NG-Test CARBA 5. Whole-genome sequencing revealed MMOR1 contained *bla*_IMP-27_, but no other carbapenemase genes. Additional testing with NG-Test CARBA 5 revealed a false-positive NDM band when the assay was overloaded with test inoculum. Supplementary isolates were tested with an overloaded inoculum (*n* = 6 M. morganii; *n* = 1 P. mirabilis; *n* = 1 IMP-27-producing *P. rettgeri*; *n* = 1 IMP-1-producing E. coli; *n* = 1 K. pneumoniae), and two non-carbapenemase-producing carbapenem non-susceptible M. morganii also generated a false-positive NDM band; though, this was not universal among this species. A dual IMP+/NDM+ M. morganii is an unusual result that should prompt additional investigation, especially in nonendemic regions and when the susceptibility profile is incompatible. IMP-27 is not detected by Xpert Carba-R but is variably detected by NG-Test CARBA 5. The microorganism inoculum used for NG-Test CARBA 5 must be carefully controlled for accurate results.

**IMPORTANCE** The detection of carbapenemase-producing carbapenem-resistant Enterobacterales (CP-CRE) is an important function of the clinical microbiology laboratory, where positive identifications have immediate implications for infection control and surveillance strategies in the inpatient setting and can inform appropriate selection of therapy among the various novel anti-CP-CRE agents. NG-Test CARBA 5 is a relatively new lateral flow assay used for detection of carbapenemases in CP-CRE. Here, we describe the characterization of a Morganella morganii isolate that generated a false-positive NDM carbapenemase detection by this assay, and perform bacterial test inoculum experiments with additional isolates to further investigate a cause of false-positive results using the NG-Test CARBA 5. While a lateral flow assay like the NG-Test CARBA 5 is a very desirable test format for clinical laboratories, there are pitfalls to avoid when performing this test and interpreting results, including recognizing an overloaded test assay, which could lead to false-positive results.

## INTRODUCTION

Carbapenem-resistant Enterobacterales (CRE) are found globally. The mechanism of carbapenem resistance in CRE may be mediated through production of carbapenemases, or other methods (1). Carbapenemase-producing CRE (CP-CRE) are of clinical and public health concern because carbapenem resistance is transmissible between organisms and patients, these organisms have limited treatment options, there is no decolonization strategy, and infections with these organisms are associated with high mortality rates (2). The five main carbapenemases of concern in CP-CRE include KPC (endemic in the USA), OXA-48-like, VIM, IMP, and NDM, whose prevalence and distribution differ by geographic region (3–5).

Clinical laboratories may employ a variety of methods to detect CP-CRE, including detection of carbapenemase enzymatic activity (e.g., carbapenem inactivation method [CIM], RAPIDEC CARBA NP), carbapenemase proteins via antibodies (e.g., NG-Test CARBA 5), or nucleic acid sequences encoding carbapenemases (e.g., Xpert Carba-R) (6). The NG-Test CARBA 5 lateral flow assay (LFA) is particularly attractive to clinical laboratories because of its ease of use, relative low cost, and ability to identify and differentiate the five main carbapenemase groups of concern. The identification of a patient harboring CP-CRE is a significant result that may prompt long-term isolation and contact precautions for the patient, and prompt screening for CP-CRE colonization in other patients to monitor for potential transmission. Hence, accurate results from these assays are important so that a patient carrying a transmissible carbapenemase is not missed (preventing transmission) and so that a patient lacking these resistance mechanisms is not unnecessarily placed on contact precautions or prompts unnecessary screening (avoiding extra costs and waste of personnel time). In addition, accurate identification of the carbapenemase type harbored by a clinical isolate (i.e., KPC, OXA-48-like, VIM, IMP, and/or NDM) can inform optimal selection of novel β-lactam/β-lactamase inhibitor combination agents for treatment of infections caused by CP-CRE, as different carbapenemases are variably inhibited by these drugs (7).

Our objective was to evaluate an isolate of Morganella morganii (MMOR1) that was initially reported by the submitting clinical laboratory as being positive for both NDM and IMP carbapenemases based on the NG-Test CARBA 5 method, in a geographic region (Missouri, USA) with a low prevalence of both resistance determinants. After the isolate was referred to our laboratory for additional work-up of these atypical results, we used a combination of methods to characterize this isolate, including phenotypic antimicrobial susceptibility testing, CIM, Xpert Carba-R, NG-Test CARBA 5, and whole-genome sequencing (WGS). Furthermore, we evaluated additional isolates on the NG-Test CARBA 5 that shared certain features in common with the MMOR1 isolate, to evaluate analytical features that might contribute to false-positive results in this test.

## RESULTS

**FIG 1:**
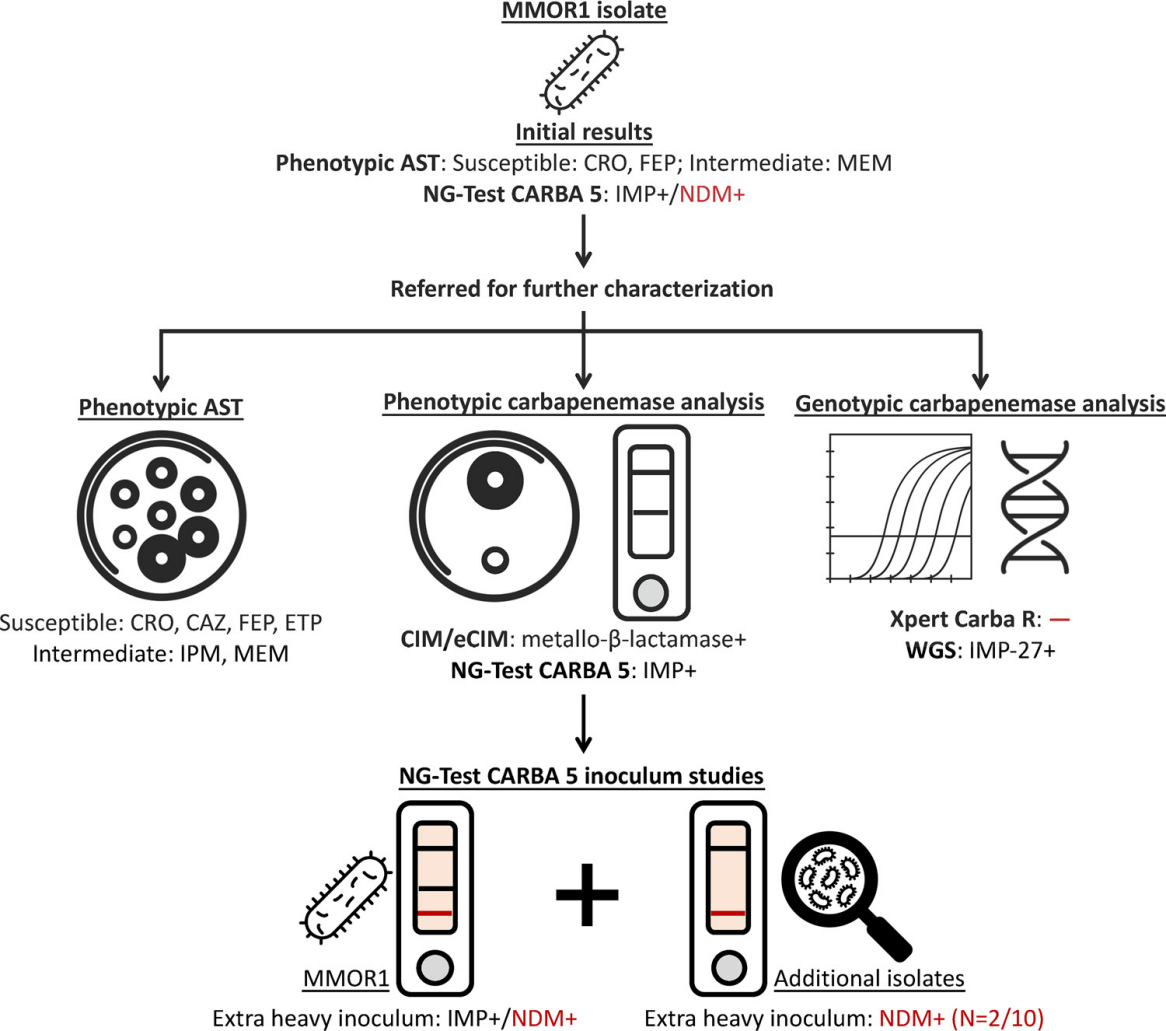
Workflow for M. morganii MMOR1 isolate evaluation and NG-Test CARBA 5 evaluation. The submitting clinical laboratory initially characterized the MMOR1 isolate by AST and the NG-Test CARBA 5, where it originally tested as IMP+/NDM+ by NG-Test CARBA 5. The isolate was referred to our facility, where follow-up testing included phenotypic methods (AST; CIM and eCIM; and repeat NG-Test CARBA 5) and genotypic methods (Xpert Carba-R and WGS). Additional testing on NG-Test CARBA 5 was performed using varying starting inoculum sizes, including an “extra heavy” inoculum of MMOR1 and other isolates sharing features in common with MMOR1. This extra heavy inoculum led to a faint false-positive NDM band (indicated in red) in MMOR1 and two of 10 additional isolates that were tested. AST, antimicrobial susceptibility testing; CRO, ceftriaxone; CAZ, ceftazidime; FEP, cefepime; ETP, ertapenem; IPM, imipenem; MEM, meropenem; CIM, carbapenem inactivation method; eCIM, CIM with EDTA; WGS, whole-genome sequencing. False results are indicated in red. Bacteria, petri dish, PCR, and DNA icons were obtained from The Noun Project (https://thenounproject.com).

An isolate of M. morganii (MMOR1) that tested susceptible to 3^rd^/4^th^-generation cephalosporins and intermediate to meropenem but positive for NDM and IMP carbapenemases by NG-Test CARBA 5 was referred to our laboratory by an outside clinical laboratory for further characterization, given the inconsistent susceptibility profile and unusual epidemiological profile for our region. Additional testing of the MMOR1 isolate and evaluation of other *Morganellaceae* with similar phenotypic or genotypic profiles on the NG-Test CARBA 5 was performed and is shown in Fig. 1.

### Antimicrobial susceptibility profile and phenotypic assessment of carbapenemase production in the MMOR1 isolate.

The M. morganii MMOR1 isolate was extensively tested with antimicrobial susceptibility testing (AST) using a combination of disk diffusion and gradient diffusion methods (Table 1). Since non-susceptibility to imipenem (a carbapenem) is not unexpected in a member of the *Morganellaceae*, the most interesting features of this isolate’s AST profile were that it tested intermediate to the carbapenem, meropenem, but susceptible to the carbapenem, ertapenem, and susceptible to 3^rd^ (ceftriaxone, ceftazidime) and 4^th^ (cefepime) generation cephalosporins. This AST profile is not consistent with typical NDM, IMP, or NDM/IMP-producing isolates, which often test resistant to these antibiotics and would be expected to confer high-level phenotypic resistance to carbapenems (8, 9). Testing via CIM and CIM with EDTA (eCIM) confirmed that the isolate was positive for carbapenemase production consistent with a metallo-β-lactamase (Table 1).

**TABLE 1:** Antimicrobial susceptibility testing profile and CIM and eCIM results of the M. morganii MMOR1 isolate^*a*^

Antimicrobial agent or test	Zone size (mm)	MIC (μg/mL)	Interpretation^*b*^
β-lactams			
Ampicillin	6		R
Ampicillin/sulbactam	13		I
Cefazolin	6		R
Cefotetan	12		R
Ceftriaxone	23		S
Ceftazidime	28		S
Cefepime	29		S
Aztreonam	36		S
Piperacillin/tazobactam	32		S
Ertapenem	23		S
Imipenem	21		I
Meropenem	22		I
Ceftazidime/avibactam	24		S
Imipenem/relebactam^*c*^		2/4	^*d*^
Meropenem/vaborbactam^*c*^		0.5/8	S
Other agents			
Gentamicin	25		S
Tobramycin	24		S
Amikacin	24		S
Trimethoprim/sulfamethoxazole	26		S
Levofloxacin	33		S
Ciprofloxacin	35		S
Minocycline	10		R
Doxycycline	6		R
CIM	6		Carbapenemase+
eCIM	26		Metallo-β-lactamase+

a MIC, minimal inhibitory concentration; CIM, carbapenem inactivation method; eCIM, CIM with EDTA; S, susceptible; I, intermediate; R, resistant; +, positive.

b Interpretations are according to the CLSI M100 32^nd^ Edition.

c Tested by gradient diffusion.

d Imipenem/relebactam breakpoints for Enterobacterales do not apply to the family *Morganellaceae*, which includes M. morganii. This result is interpreted as “intermediate” for other Enterobacterales.

### Carbapenemase testing and genomic analysis of the M. morganii isolate.

**FIG 2:**
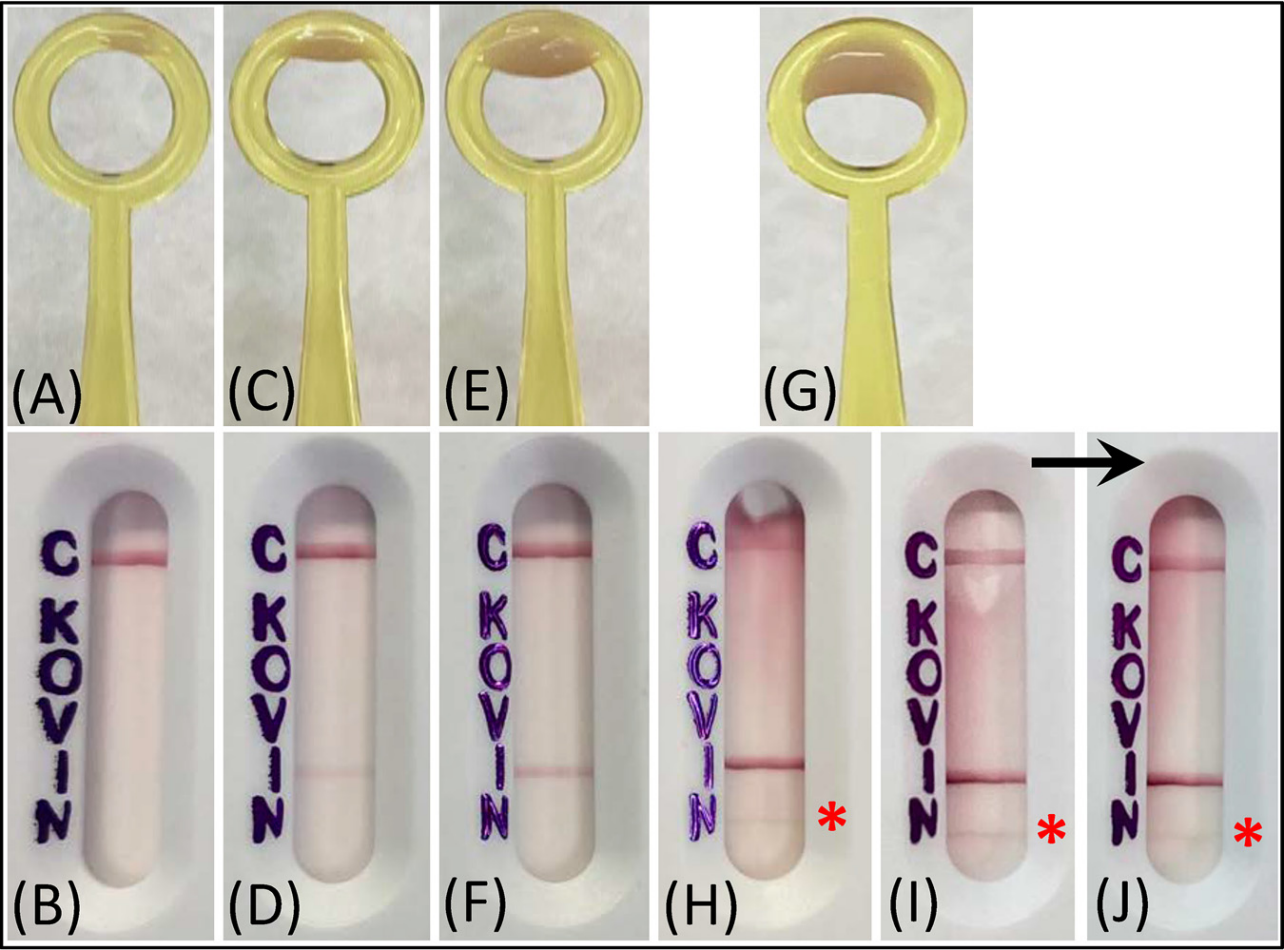
NG-Test CARBA 5 results for the MMOR1 isolate tested with different inoculum sizes. The NG-Test CARBA 5 lateral flow assay was performed according to the package insert, or the test inoculum was varied; representative images of inoculum and corresponding results are shown. (A and B) light inoculum (touch 1 area of growth with a 10-μL loop), (C and D) standard inoculum (touch 3 areas of growth with a 10-μL loop), (E and F) heavy inoculum (touch 6 areas of growth with a 10-μL loop), (G and H) extra heavy inoculum (1/2 of a 10-μL loop). (I) extra heavy inoculum tested on a separate day with a different lot number of NG-Test CARBA 5 (biological replicate). (J) same test cartridge in panel (I) after several minutes extra incubation (i.e., read beyond the standard 15 min incubation time, to demonstrate how the cartridge background becomes more “typical” appearing if the test is read after the correct incubation window). The red asterisk highlights the false-positive NDM band that appeared when an extra heavy inoculum was tested.

For detection of the five most common carbapenemase groups occurring in Enterobacterales, the isolate was tested by Xpert Carba-R and retested with the NG-Test CARBA 5 assay. The MMOR1 isolate tested negative for all carbapenemase targets by Xpert Carba-R, but positive for (only) IMP by repeat NG-Test CARBA 5 (Fig. 2). Due to the discrepancy in IMP detection by these assays, and since neither assay detected the NDM that was originally detected by the submitting clinical laboratory, the isolate was subjected to WGS analysis, which revealed the presence of the *bla*_IMP-27_ carbapenemase gene and confirmed the absence of *bla*_NDM_.

### NG-Test CARBA 5 inoculum studies.

To further investigate the false-positive NDM result that was originally reported by the submitting clinical laboratory for MMOR1 based on the NG-Test CARBA 5, additional experiments were conducted with this isolate to understand whether variations in the starting test inoculum could perturb assay performance. When less starting inoculum than recommended in the package insert was used (light inoculum), the NDM band was absent, and the IMP band became so faint it was nearly undetectable. Doubling the amount of starting inoculum (heavy inoculum) produced only a well-defined IMP band (Fig. 2). When the assay was grossly overloaded (extra heavy inoculum) several things occurred: the LFA flowed poorly, usually taking the full 15 min incubation for the control band to appear; the strip background did not properly clear of pink color; and a faint band appeared in the NDM region of the test cartridge. This result was reproducible across different LFA lots tested on different days (false-positive NDM band present in 3 of 3 biological replicates; 2 are shown in Fig. 2). With extra incubation time (between 2 and 7 min longer than recommended in the package insert), the background color of the LFA began to clear, and the overloaded test cartridges began to look more “typical.”

**FIG 3:**
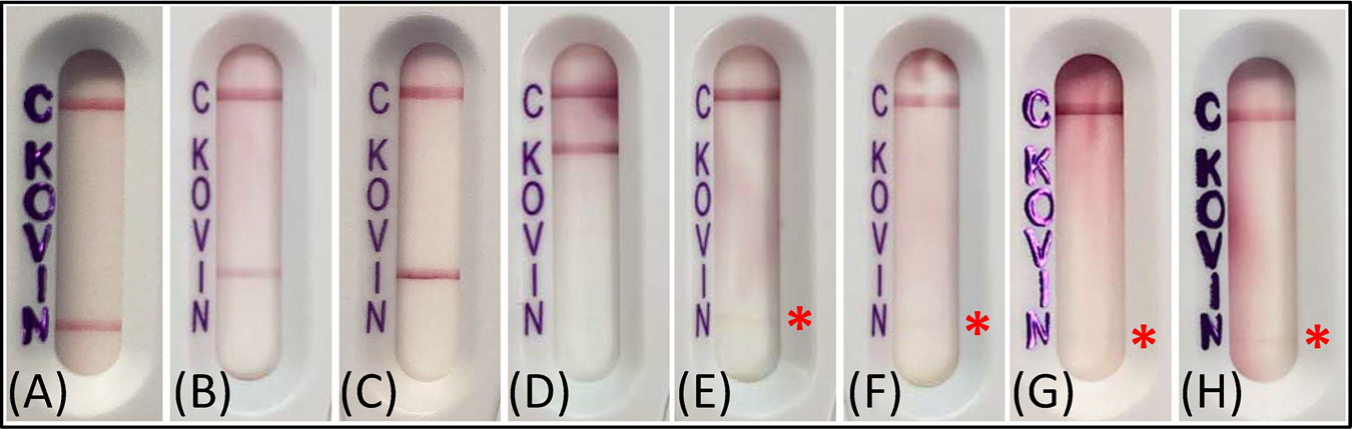
Representative NG-Test CARBA 5 results for other isolates tested with an “extra heavy” inoculum. Additional isolates were tested with an extra heavy inoculum (1/2 of 10-μL loop) on NG-Test CARBA 5, and the following representative examples are shown: (A) NDM-positive K. pneumoniae ATCC BAA-2146 (standard inoculum testing of a true-positive NDM isolate is shown for visual comparison to other LFAs), (B) IMP-positive E. coli NCTC 13476, (C) IMP-27-positive PR1, (D) KPC-positive MMOR6, (E) non-carbapenemase-producing MMOR5, and (F) technical replicate of MMOR5, (G) non-carbapenemase-producing MMOR3, and (H) technical replicate of MMOR3. The red asterisk highlights the false-positive NDM band that appeared in two of 10 other isolates tested with an extra heavy inoculum.

To understand whether the false-positive NDM band was specific to the MMOR1 isolate or an error that broadly occurs with inoculum overloading, additional carbapenem non-susceptible isolates were tested. Pertinent β-lactam resistance profile information, CIM result, carbapenemase gene detection, and the NG-Test CARBA 5 result for each additional isolate tested at the standard inoculum and with an extra heavy inoculum are shown in Table 2. LFAs from representative isolates tested with an extra heavy inoculum on NG-Test CARBA 5 are shown in Fig. 3. The IMP-27+ PR1 isolate—which tested negative for IMP on Xpert Carba-R but positive for IMP on NG-Test CARBA 5—did not yield a false-positive NDM band on the LFA when an extra heavy inoculum was tested. Overall, two of the 10 isolates tested yielded a faint false-positive NDM band; both were noncarbapenemase-producing M. morganii isolates (MMOR3, MMOR5), and the false-positive band was reproducible across different LFA lots (2 of 2 technical replicates positive each, for MMOR3 and MMOR5).

**TABLE 2:** β-lactam resistance profiles, CIM results, Xpert Carba-R results, and NG-Test CARBA 5 results of other tested isolates^*a*^

Test isolate	β-lactam resistance		Xpert Carba-R	NG-Test CARBA 5 result	
		CIM result	result	Standard inoculum	Extra heavy inoculum
M. morganii MMOR2	CRO (R), CAZ (R), FEP (S), ETP (S), IPM (R), MEM (S)	Negative	Negative	Negative	Negative
M. morganii MMOR3	CRO (R), CAZ (S), FEP (S), ETP (S), IPM (I), MEM (S)	Negative	Negative	Negative	NDM^*b*^
M. morganii MMOR4	CRO (I), CAZ (R), FEP (S), ETP (S), IPM (R), MEM (S)	Negative	Negative	Negative	Negative
M. morganii MMOR5	CRO (S), CAZ (I), FEP (S), ETP (S), IPM (I), MEM (S)	Negative	Negative	Negative	NDM^*b*^
M. morganii MMOR6	CRO (R), CAZ (S), FEP (S), ETP (S), IPM (R), MEM (I)	Positive	KPC	KPC	KPC
M. morganii MMOR7	CRO (R), CAZ (R), FEP (R), ETP (R), IPM (R), MEM (R)	Positive	KPC	KPC	KPC
P. mirabilis PMIR1	CRO (S), CAZ (S), FEP (SDD), ETP (S), IPM (R), MEM (I)	Positive	KPC	KPC	KPC
K. pneumoniae ATCC BAA-1706	Negative NG-Test CARBA 5 CRE control	Negative	Negative	Negative	Negative
E. coli NCTC 13476 (IMP-1+)	IMP+ NG-Test CARBA 5 CRE control	Positive	IMP	IMP	IMP
*P. rettgeri* PR1 (IMP-27+)	CRO (R), CAZ (S), FEP (SDD), ETP (R), IPM (R), MEM (R)	Positive	Negative	IMP	IMP

a CRO, ceftriaxone; CAZ, ceftazidime; FEP, cefepime; ETP, ertapenem; IPM, imipenem; MEM, meropenem; S, susceptible; SDD, susceptible dose dependent; I, intermediate; R, resistant.

b Indicates a faint false-positive NDM band.

## DISCUSSION

Our laboratory encountered a M. morganii clinical isolate (MMOR1) that was initially characterized by the submitting clinical laboratory as dual IMP+/NDM+ by NG-Test CARBA 5—an unusual result (particularly in our region) that should prompt additional investigation, especially if the AST profile is inconsistent with the carbapenemase test result. Upon further evaluation, we found that MMOR1 was an IMP-27-producing isolate that tested positive for an IMP band on the NG-Test CARBA 5, but also generated a weak, but reproducible, false-positive NDM band in the assay when the starting test inoculum was too heavy. Interestingly, M. morganii isolates were not included as test organisms in the clinical trials for the NG-Test CARBA 5 device (10). Few published studies have included this organism, and even fewer studies have tested more than one isolate of this species with this test (11–18). We tested six M. morganii isolates at both the package insert-recommended test inoculum as well as an extra heavy test inoculum and observed that two non-carbapenemase-producing M. morganii isolates generated a faint false-positive NDM band when the assay was overloaded. Several other species with and without carbapenemases were tested, though none generated this faint false-positive NDM band. As such, this false reactivity did not seem to be specifically associated with IMP/IMP-27 production or carbapenemase-producing *Morganellaceae*, or universal to all M. morganii. A potential explanation for faint NDM false-positivity is that some M. morganii produce low quantities of a protein that shares homology with the NDM epitopes detected by NG-Test CARBA 5. It is also possible that the NDM band is more likely to be affected when the assay is performed incorrectly simply because it is the first line the sample crosses during migration through the LFA.

The NG-Test CARBA 5 LFA is FDA-cleared for testing colonies of carbapenem nonsusceptible Enterobacterales and Pseudomonas aeruginosa. A prior study showed that this assay is highly sensitive and specific for detection of the five most common carbapenemases produced by these organisms (19). Other studies have reported false-positive results for the assay. Zhu et al. reported an IMP-4-producing Enterobacter cloacae that generated an IMP band and a weak false-positive NDM band on NG-Test CARBA 5 (20). Hopkins et al. reported an NDM-producing isolate that generated a false-positive VIM band, and a dual carbapenemase-producing isolate that generated a false-positive KPC band—though these results were not reproducible (15). Kon et al. reported a SME-producing Serratia marcescens that generated a false-positive OXA-48-like band (17). These studies did not speculate on the cause of the false-positive bands. In the study by Zhu et al. the inoculum was described as a 1-μL loopful of bacteria, while in Hopkins et al. and Kon et al. the inoculum was described as one colony (15, 17, 20). A 1-μL loopful would not constitute an “extra heavy” inoculum; however, since bacterial colonies vary widely in size, it is possible that Hopkins et al. or Kon et al. overloaded test inoculum in some of their LFAs. One commonality across all studies (the present study included) is that the species yielding false-positive results are chromosomal AmpC-producing Enterobacterales. In a different study by Lee et al., though not cleared for this use, it was observed that NG-Test CARBA 5 (and the related assay NG-Test CTX-M MULTI) generated false-positive bands for all targets when a positive blood culture broth (which grew a non-carbapenemase-producing E. coli) and serum from a particular patient were tested (21). The authors postulated that the patient’s blood/serum contained endogenous anti-mouse antibodies directed against the antibodies used in NG-Test CARBA 5, causing false-positive bands in the assay.

While LFAs such as the NG-Test CARBA 5 are a very desirable test format for the clinical laboratory due to their typical ease of use, rapid time to results, and relatively low cost per test, there are several pitfalls to avoid. Careful preparation of the inoculum/sample size is critical, as heavily overloading the LFA can lead to results that are difficult to interpret or even false-positive. General indications of an overloaded LFA include slower sample advancement than is typical for the assay and failure of the test strip background to clear of most/all color. Another critical factor is cartridge incubation time, as extended incubations (i.e., longer than the correct reading window for the test) may lead to incorrect interpretation of results because the test strip starts to look more “normal” (e.g., clearer background with the presence of control line). It is essential to always perform, read, and interpret these tests according to the manufacturer’s instructions. Overall, an overloaded test inoculum is likely to occur in the clinical environment given the present clinical case and that the “extra heavy” inoculum is not unreasonably large, especially considering the relatively large size of Enterobacterales colonies. Since the package insert indicates that the test user should “touch 3 colonies with a loop” to obtain the test inoculum, addition of images to the manufacturer package insert to demonstrate what “too little,” “correct,” and “too much” inoculum look like is advised, so that the user can visualize the average correct inoculum.

Interestingly, IMP-27 is a known testing limitation of Xpert Carba-R and is variably detected by NG-Test CARBA 5 (12, 22–24). Our lab previously identified IMP-27 in a *P. rettgeri* isolate (PR1) from a patient in Missouri (22). In the current study, once the NDM result was confirmed as false-positive and the M. morganii isolate was determined to carry IMP-27, the AST profile of the isolate made more sense. Though not a common carbapenemase variant reported in CRE, IMP-27 is more commonly reported among members of the *Morganellaceae* (at least in human clinical isolates), and some of these isolates test more broadly susceptible to the β-lactams, including to ceftazidime, cefepime, ertapenem, and meropenem (12, 22–25). While most of the IMP-27+ isolates have been reported as ceftriaxone or cefotaxime intermediate or resistant, MMOR1 tested susceptible to ceftriaxone, though right at the breakpoint (23 mm). Given the typical precision window of AST, the isolate may have tested intermediate to ceftriaxone upon repeat testing.

Our study demonstrates the necessity of correlating results from assays that target specific carbapenemases with the phenotypic AST profile of the isolate and local epidemiology; if these are not in alignment, extra testing is warranted. Our study also illustrates that reliance on assays that target specific carbapenemase variants for detection of carbapenemase-producing isolates, such as Xpert Carba-R, may miss IMP-27-producing isolates. Hence, laboratories should consider a complementary approach to carbapenemase detection as the ideal. Phenotypic methods that identify carbapenemases (such as CIM) are an important complementary component of a carbapenemase detection workflow, as a non-targeted approach to detect the activity of these enzymes is less subject to false-negative results due to variations in protein or nucleic acid sequence.

## MATERIALS AND METHODS

### Clinical isolates.

Bacterial isolates tested in this study were obtained from the Barnes-Jewish Hospital (BJH) clinical microbiology laboratory in St. Louis, MO, USA—either recovered from clinical specimens or referred by another clinical laboratory to BJH for further testing (MMOR1 isolate) as part of routine clinical care. The Providencia rettgeri PR1 isolate was described in a previous study (22). Clinical isolates were identified by MALDI-TOF MS (MALDI Biotyper, Bruker Daltonics, Billerica, MA). This study was granted a “not human subjects research” determination by the IRB committee from Washington University in St. Louis (IRB no. 202212020).

After routine clinical testing at BJH (including antimicrobial susceptibility testing, CIM, and Xpert Carba-R) isolates were stored in skim milk (BD, Franklin Lakes, NJ) at −80°C. For NG-Test CARBA 5 testing, dual CIM and eCIM testing, and whole-genome sequencing, isolates were subcultured from frozen stocks onto blood agar plates (Hardy Diagnostics, Santa Maria, CA) with a meropenem disk (BD) to maintain selective pressure and incubated overnight. All isolates were serially subcultured twice with meropenem prior to testing.

### Antimicrobial susceptibility testing.

The following CLSI recommended quality control (QC) strains from the American Type Culture Collection (ATCC, Manassas, VA) were used: Escherichia coli ATCC 25922, Pseudomonas aeruginosa ATCC 27853, and Staphylococcus aureus ATCC 25923 (26). Kirby-Bauer disk diffusion with amikacin, ampicillin, ampicillin/sulbactam, aztreonam, cefazolin, cefepime, cefotetan, ceftazidime, ceftazidime/avibactam (Hardy Diagnostics), ceftriaxone, ciprofloxacin, doxycycline, ertapenem, gentamicin, imipenem, levofloxacin, meropenem, minocycline, piperacillin/tazobactam, tobramycin, and trimethoprim/sulfamethoxazole (all disks were from BD unless otherwise indicated) was performed and interpreted in accordance with CLSI guidelines (26). Gradient diffusion with imipenem/relebactam (bioMérieux, Durhman, NC) and meropenem/vaborbactam (Liofilchem, Waltham, MA) was performed and interpreted in accordance with package inserts and CLSI guidelines (26). Mueller-Hinton (MH) II agar (BD) was used for both AST methods.

### Carbapenem inactivation method (CIM), and CIM with EDTA (eCIM).

The following CLSI recommended QC strains were used: E. coli ATCC 25922, Klebsiella pneumoniae ATCC BAA-1705 (KPC+), K. pneumoniae ATCC BAA-1706 (non-carbapenemase-producing), and K. pneumoniae ATCC BAA-2146 (NDM+) (26). The CIM and eCIM assays for detection of any carbapenemase production or metallo-β-lactamase production, respectively, were performed and interpreted as described in McMullen et al. (27).

### GeneXpert Xpert Carba-R.

The Xpert Carba-R (Cepheid, Sunnyvale, CA) real-time PCR assay for detection of KPC, OXA-48-like, VIM, IMP, and NDM carbapenemase genes was performed according to the manufacturer’s instructions (27). Clinical isolates positive for the following were used for QC: an NDM-1+ Enterobacter cloacae, a KPC+ K. pneumoniae, a VIM+ P. aeruginosa, an OXA-48+ K. pneumoniae, and an IMP+ E. cloacae.

### NG-Test CARBA 5.

The following manufacturer recommended QC isolates from ATCC and the UK Health Security Agency (NCTC isolates) were used: K. pneumoniae ATCC BAA-1705 (KPC+), K. pneumoniae ATCC BAA-2146 (NDM+), K. pneumoniae ATCC BAA-1706 (non-carbapenemase-producing), K. pneumoniae NCTC 13442 (OXA-48+), K. pneumoniae NCTC 13439 (VIM+), and E. coli NCTC 13476 (IMP+). The NG-Test CARBA 5 (NG Biotech, Guipry, France) LFA for detection of KPC, OXA-48-like, VIM, IMP, and NDM carbapenemases was performed both according to the manufacturer’s instructions and with modifications to the starting test inoculum (28). To perform the assay according to the manufacturer’s instructions for each isolate (standard inoculum): 5 drops (150 μL) of test extraction buffer was added to a microtube provided in the kit; a 10-μL loop was used to touch 3 areas of growth from around the meropenem disk (representing the package insert instructions to “touch 3 colonies with a loop”) to ensure only growth under carbapenem-selective pressure was used to avoid carbapenem resistance gene loss, which was then resuspended in the extraction buffer. The microtube was vortexed for 10 s, then the transfer pipette provided in the kit was used to transfer 100 μL of mixture to the LFA cartridge. The test was incubated at room temperature for 15 min before reading. For test interpretation, one red line must appear in the control region for a valid test, and while the intensity of any of the 5 test lines may vary, a weak line is still a positive result for a given target.

Experiments using modified starting inoculum for MMOR1 were performed as described above, except that non-standard amounts of growth from around the meropenem disk were obtained with a 10-μL loop and used in the assay, including: light inoculum (touch 1 area of growth), heavy inoculum (touch 6 areas of growth), or extra heavy inoculum (1/2 of a 10-μL loopful of growth). Representative images of each inoculum size are shown in Fig. 2. Extra heavy inoculum experiments were repeated on different days with different test lot numbers, for 2 biological replicates (3 total replicates). The extra heavy inoculum LFAs were also held several minutes beyond the standard 15 min incubation time (between +2 to +7 min), to observe test strip background and line appearance if the test cartridge was read after the designated reading window. Other clinical isolates were tested according to the manufacturer’s instructions and with an extra heavy test inoculum. Any isolate generating a false-positive NDM band was retested on the same day for a technical replicate.

### Whole-genome sequencing.

Total genomic DNA was extracted from the MMOR1 isolate using the QIAamp BiOstic bacteremia DNA kit (Qiagen, Germantown, MD) according to the manufacturer’s instructions. A total of 0.5 ng of DNA was used to create Illumina sequencing libraries with a modification of the Nextera XT DNA library preparation kit (Illumina, San Diego, CA) (29). The sample was sequenced using the Illumina NovaSeq 6000 platform, operated by the Genome Technology Access Center at Washington University School of Medicine, to obtain ~2,000,000 reads (2 ×150 bp). Forward and reverse reads had adapter content removed with Trimmomatic v0.36 (30) and were assembled into a draft genome assembly using Unicycler v0.4.7 (31). The scaffold.fasta file was submitted to the ResFinder webserver in November 2021 (32).

### Data availability.

The MMOR1 draft whole-genome sequence that is new as part of this work was deposited to NCBI under BioProject PRJNA932828.
